# Mechanistic target of rapamycin complex 1 signaling regulates cell proliferation, cell survival, and differentiation in regenerating zebrafish fins

**DOI:** 10.1186/s12861-014-0042-9

**Published:** 2014-12-06

**Authors:** Kentaro Hirose, Taishi Shiomi, Shunya Hozumi, Yutaka Kikuchi

**Affiliations:** Department of Biological Science, Graduate School of Science, Hiroshima University, Kagamiyama 1-3-1, Higashi-Hiroshima, Hiroshima 739-8526 Japan

**Keywords:** Mechanistic target of rapamycin, Fin, Regeneration, Zebrafish, Osteoblast, Cell proliferation, Cell survival, Differentiation

## Abstract

**Background:**

The mechanistic target of rapamycin complex1 (mTORC1) signaling pathway has been implicated in functions of multicellular processes, including cell growth and metabolism. Although recent reports showed that many signaling pathways, including Activin, Bmp, Fgf, sonic hedgehog, Insulin-like growth factor (IGF), Notch, retinoic acid, and Wnt, are implicated in non-mammalian vertebrate regeneration, also known as epimorphic regeneration, mTORC1 function remains unknown.

**Results:**

To investigate the role of mTORC1 signaling pathway in zebrafish caudal fin, we examined the activation and function of mTORC1 signaling using an antibody against phosphorylated S6 kinase and a specific inhibitor, rapamycin. mTORC1 signaling is activated in proliferative cells of intra-ray and wound epidermal cells before blastema formation, as well as in proliferative blastema cells, wound epidermal cells, and osteoblasts during regenerative outgrowth. Before blastema formation, proliferation of intra-ray and wound epidermal cells is suppressed, but cell death is not affected by mTORC1 signaling inhibition with rapamycin. Moreover, rapamycin treatment inhibits blastema and wound epidermal cell proliferation and survival during blastema formation and regenerative outgrowth, as well as osteoblast proliferation and differentiation during regenerative outgrowth. We further determined that mTORC1 signaling is regulated through IGF-1 receptor/phosphatidylinositol-3 kinase and Wnt pathways during fin regeneration.

**Conclusion:**

Taken together, our findings reveal that mTORC1 signaling regulates proliferation, survival, and differentiation of intra-ray cells, wound epidermis, blastema cells, and/or osteoblasts in various fin regeneration stages downstream of IGF and Wnt signaling pathways.

**Electronic supplementary material:**

The online version of this article (doi:10.1186/s12861-014-0042-9) contains supplementary material, which is available to authorized users.

## Background

Mammalians present a limited ability for organ regeneration, whereas various non-mammalian vertebrates such as teleosts and urodele amphibians show outstanding regeneration ability. Among them, the zebrafish is a useful animal model, which has been used to study the regeneration of several organs or appendages [1,2]. The adult zebrafish caudal fin is composed of multiple cell types, including fibroblast-like mesenchymal cells, osteoblasts, endothelial cells, neurons, and epidermal cells, and the fin regeneration process presents three stages: pre-blastema formation, blastema formation, and regenerative outgrowth [3-5]. Following fin amputation, epidermal cells migrate to cover the wound within 12 hours post amputation (hpa) [5]. The intra-ray mesenchymal cells and osteoblasts then migrate toward the amputation plane by 24 hpa (pre-blastema formation stage) [5]. From 18 to 24 hpa, these intra-ray mesenchymal cells and osteoblasts begin to proliferate [3] and, as a result, a population of these cells, named blastema, is formed underneath the wound epidermis by 48 hpa (blastema formation stage). After 48 hpa, regenerative outgrowth starts and the ray blastema mainly consists of three distinct domains: the distal blastema, proliferative zone, and differentiation zone (72 hpa) [5,6]. The distal blastema barely contains proliferative blastema cells, and the proliferative zone contains highly proliferative mesenchymal cells (the proximal medial blastema) and osteoblasts (72 hpa) [3,5,6].

Since rapamycin presents various physiological functions such as antifungal, immunosuppressive, and antiproliferative properties, many researchers have focused on the identification of rapamycin intracellular targets [7]. Mechanistic target of rapamycin (mTOR), a serine/threonine kinase, has been shown to be a rapamycin target in yeast, and most eukaryotes have this protein [7,8]. The mTOR signaling pathway is mainly involved in cell growth and metabolism as two distinct complex types, mTOR complex 1 (mTORC1) and 2 (mTORC2) [7,8]. The mTORC1 signaling pathway is involved in multicellular processes, including protein synthesis, lipid synthesis, glycolysis, and autophagy, and is specifically inhibited by rapamycin [7,8]. mTORC1 signaling is known to regulate protein synthesis mainly through direct phosphorylation of S6 kinase (S6K) [7,8].

Many signaling pathways, including Activin, Bmp, Fgf, sonic hedgehog, Insulin-like growth factor (IGF), Notch, retinoic acid, Wnt, and reactive oxygen species (ROS), are implicated in the regulation of cell proliferation and/or differentiation in non-mammalian vertebrate regeneration, also known as epimorphic regeneration [2,9-12]. However, the spatiotemporal activation and function of the mTORC1 signaling pathway during epimorphic regeneration remains unknown. In this study, we explored the activation and function of mTORC1 signaling during various stages of zebrafish caudal fin regeneration, and identified the upstream signaling pathway leading to mTORC1 signaling activation during caudal fin regeneration.

## Results

### Spatiotemporal dynamism of mTORC1 signaling activation during fin regeneration

**Figure 1:**
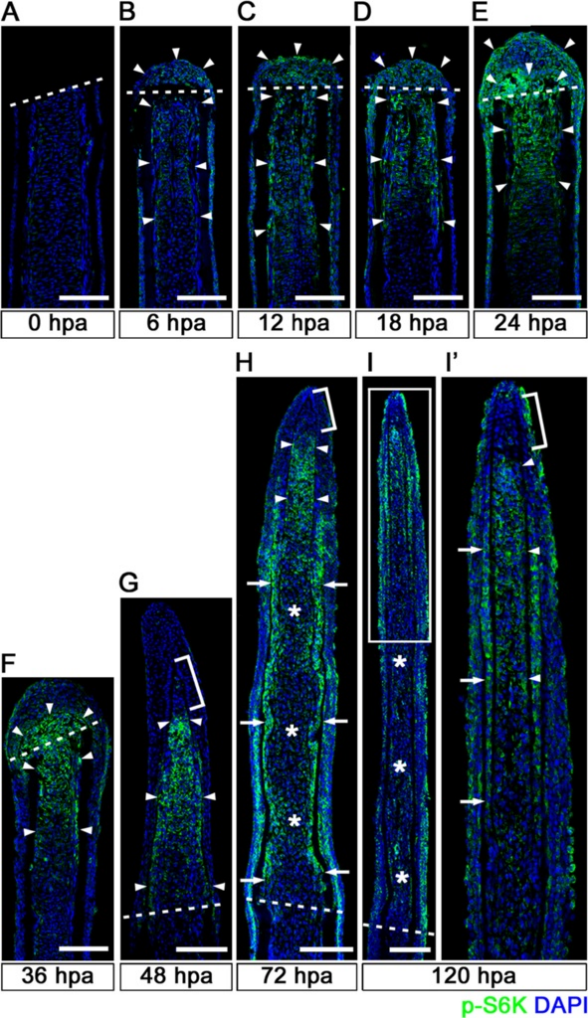
**Spatiotemporal activation of S6K during zebrafish fin regeneration. (A-I’)** Longitudinal sections of wild-type fin regenerates that were immunohistochemically stained with an antibody against p-S6K (green) at 0 **(A)**, 6 **(B)**, 12 **(C)**, 18 **(D)**, 24 **(E)**, 36 **(F)**, 48 **(G)**, 72 **(H)**, and 120 **(I,I’)** hpa (0 hpa, n = 3; 6 hpa, n = 4; 12 hpa, n = 3; 18 hpa, n = 4; 24 hpa, n = 5; 36 hpa, n = 4; 48 hpa, n = 3; 72 hpa, n = 4; 120 hpa, n = 3). The boxed area in I is enlarged in I’. DAPI fluorescent signal (blue) indicates the presence of nuclei. Dashed white lines indicate the amputation plane. The p-S6K fluorescent signals were barely detectable in the amputated fin at 0 hpa **(A)**. At 6 hpa, p-S6K-positive cells were found in both intra-ray and epidermal cells (arrowheads in **B**), and the number of p-S6k-positive cells increased by 24 hpa (arrowheads in **C**, **D**, and **E**). Although p-S6K-positive cells were found in both the blastema and intra-ray region adjacent to and proximal to the amputation plane at 36 hpa (arrowheads in **F**), p-S6K-positive cells were mainly detected in the blastema at 48 hpa (arrowheads in **G**). At 72 and 120 hpa, p-S6K-positive cells were observed in the bilateral strip regions (arrows in **H** and **I’**), in the putative proximal medial blastema (arrowheads in **H** and **I’**), and in the wound epidermis, but not in the putative differentiated blastema cells (asterisks in **H** and **I**). Brackets indicate the p-S6K-negative cells in the tip of the putative proximal medial blastema domain and putative distal blastema **(G,H,I’)**. It should be noted that both p-S6K and DAPI fluorescent signals did not overlap, as p-S6K and genomic DNA (DAPI specifically stains double-strand DNA) are localized in the cytosol and nucleus, respectively. Scale bars: 100 μm.

To investigate the molecular mechanisms of regeneration, we analyzed the signaling pathways involved in zebrafish fin regeneration using various inhibitors and drugs. Our experiments indicated that rapamycin, a well-known inhibitor of mTORC1 signaling, presented a strong inhibitory effect on fin regeneration. To analyze the activation of mTORC1 signaling during fin regeneration, spatiotemporal distribution of phosphorylated S6 kinase (p-S6K), an activated form of S6K, was first examined by immunohistochemistry. Although no p-S6K-positive cells were found in intra-ray and epidermal cells immediately after fin amputation (0 hpa) (Figure 1A), p-S6K signals were detected as early as 6 hpa (Figure 1B). The number of p-S6K-positive cells in the intra-ray, epidermis, and blastema was markedly increased, and p-S6K-positive cell localization gradually changed with the progression of fin regeneration. These p-S6K-positive cells were widely distributed in the intra-ray and wound epidermis proximal to the amputation plane from 6 to 18 hpa (arrowheads in Figure 1B-D). From 24 hpa, these cells started to accumulate underneath the wound epidermis, and p-S6K-positive blastema cells were evident at 36 hpa (arrowheads in Figure 1E,F). After 48 hpa, p-S6K-positive cells were mainly detected in the blastema and wound epidermis. At 72 and 120 hpa, p-S6K signals were restricted to three distinct domains in the blastema, the putative proximal medial blastema domain (arrowheads in Figure 1H,I’) [3], the bilateral stripped-domains (arrows in Figure 1H,I’), where differentiating osteoblasts and their progenitor cells are localized [11,13], and the wound epidermis. On the other hand, it is interesting to note that p-S6K signals were absent in the tip of the putative proximal medial blastema domain and putative distal blastema (brackets in Figure 1G,H,I’). These results suggest that, although the mTORC1 signaling pathway is widely activated in the intra-ray, wound epidermal cells, and blastema cells until 48 hpa, mTORC1 signaling is gradually restricted to the putative proliferative blastema cells and osteoblasts after 72 hpa.

**Figure 2:**
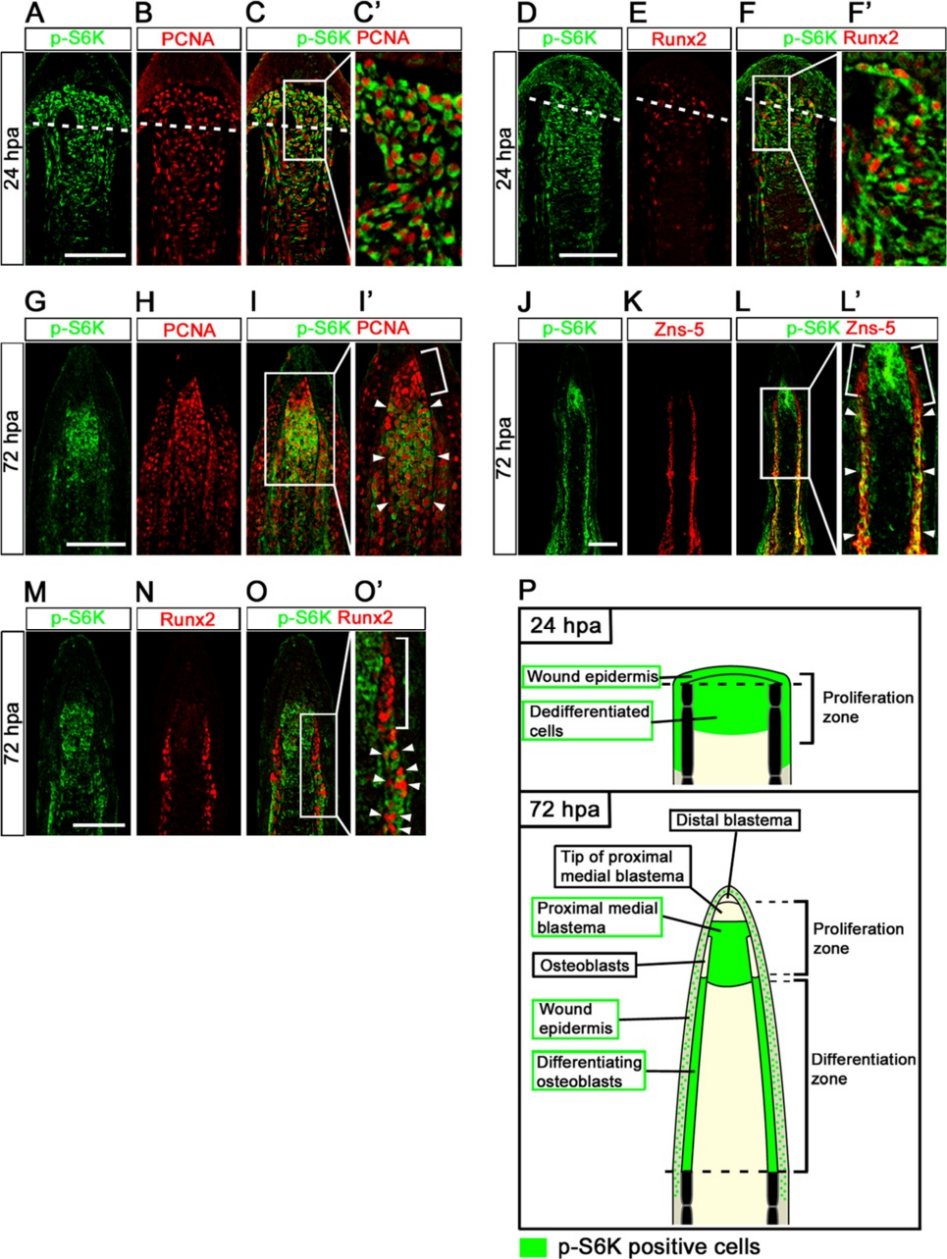
**Distributions of S6K**-**positive cells and proliferative cells at 24 and 72 hpa. (A-F’)** Longitudinal sections of wild-type fin regenerates that were co-immunohistochemically stained with antibodies against p-S6K (green) and PCNA (red) (**A-C’**, n = 3) or p-S6K (green) and Runx2 (red) (**D-F’**, n = 3) at 24 hpa. p-S6K positive cells were PCNA- or Runx2-positive at 24 hpa **(C’,F’)**. The boxed areas in **C** and **F** are enlarged in **C’** and **F’**, respectively. Dashed white lines indicate the amputation planes. **(G-O’)** Longitudinal sections of wild-type fin regenerates that were co-immunostained with antibodies against p-S6K (green) and PCNA (red) (**G-I’**, n = 5), p-S6K (green) and Zns-5 (red) (**J-L’**, n = 3), or p-S6K (green) and Runx2 (red) (**M-O’**, n = 4) at 72 hpa. PCNA-positive cells were p-S6K-positive in the putative proximal medial blastema domain (arrowheads in **I’**), but not in the tip of the putative proximal medial blastema domain (a bracket in **I’**). Zns-5- or Runx2-positive cells in the proximal lateral blastema were also p-S6K-positive (arrowheads in **L’** and **O’**), but cells in the distal region of the lateral blastema were not (brackets in **L’** and **O’**). It should be noted that both p-S6K and PCNA or Runx2 fluorescent signals did not overlap, because p-S6K and PCNA or Runx2 are localized in the cytosol and nucleus, respectively. Scale bars: 100 μm. **(P)** Cartoon summarizing the anatomical structures of fin regenerates in longitudinal cross-sections and localization of p-S6K-positive cells in the fin regenerates at 24 and 72 hpa. Dashed lines indicate the amputation planes.

To characterize p-S6K-positive cells during the pre-blastema formation (24 hpa) and regenerative outgrowth stages (72 hpa), the fin regenerates were co-immunostained with proliferating cell nuclear antigen (PCNA), a marker for proliferative cells [14]; Zns-5, a marker for all osteoblasts independent of differentiation stages [15]; or Runx2, an osteoblast progenitor marker [16]. At 24 hpa, almost all p-S6K-positive intra-ray and blastema cells were PCNA-positive (Figure 2A-C’), and all Runx2-positive osteoblast progenitors were p-S6K-positive (Figure 2D-F’), suggesting that mTORC1 signaling is active in proliferative cells and osteoblast progenitors during the pre-blastema formation stage. At 72 hpa, PCNA-positive cells in the putative proximal medial blastema domain were p-S6K-positive (arrowheads in Figure 2I’), except that the tip of the putative proximal medial blastema domain (a bracket in Figure 2I’) was p-S6K-negative. In the bilateral-stripped domains, Zns-5- or Runx2-positive cells were p-S6K-positive (arrowheads in Figure 2L’,O’), except in the most distal regions of these domains (brackets in Figure 2L’,O’). A recent report showed that Runx2-positive self-renewing preosteoblasts are localized in the most distal region and that Runx2/Sp7 (Osterix) double-positive cells are differentiating osteoblasts [11]. Our results suggest that mTORC1 signaling is active in proliferative blastema cells and differentiating osteoblasts during the regenerative outgrowth stage. These spatiotemporal mTORC1 activation patterns prompted us to further analyze the function of mTORC1 signaling in the pre-blastema formation, blastema formation, and regenerative outgrowth stages.

### mTORC1 signaling is required for cell proliferation, but not for cell survival during the pre-blastema formation stage

**Figure 3:**
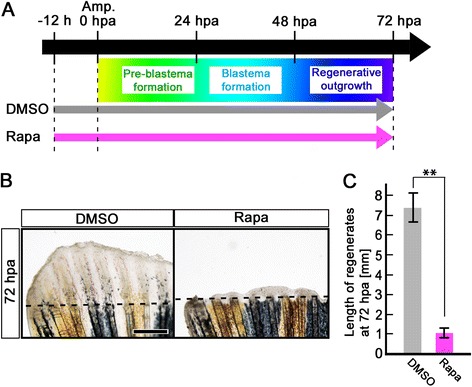
**Rapamycin treatment inhibits fin regeneration until 72 hpa. (A)** Scheme of rapamycin treatment from – 12 h to 72 hpa. **(B, C)** Rapamycin treatment significantly inhibited fin regeneration from – 12 h to 72 hpa (pre-blastema formation, blastema formation, and regenerative outgrowth stages), when compared to DMSO treatment. Dashed lines indicate the amputation planes. ** *p* < 0.01 by Student’s *t*-test. Error bars represent the standard error of 4 independent experiments. Scale bars: 500 μm in **B**.

To examine the function of mTORC1 signaling during fin regeneration, adult zebrafish were treated with a specific mTORC1 inhibitor, rapamycin, 12 h before amputation (-12 h) to 72 hpa (Figure 3A). Rapamycin significantly inhibited fin regeneration compared to DMSO (Figure 3B,C). In addition to rapamycin, we examined the two different pharmacological inhibitors, Torin1 [17] and AZD8055 [18], in mTOR signaling inhibition, and found that fin regeneration was also significantly inhibited by both Torin1 and AZD8055 treatment (Additional file 1: Figure S1). mTORC1 signaling inhibition, by rapamycin, Torin1, and AZD8055 was confirmed by the loss of p-S6K signal at 72 hpa (Additional file 2: Figure S2). The p-S6K signals were markedly reduced by 3 h treatment with these inhibitors (rapamycin, Torin1, and AZD8055) and were nearly diminished by 6 h treatment (Additional file 3: Figure S3), suggesting the specificity of p-S6K as readout of mTORC1 signaling. Furthermore, fin regeneration was also inhibited by the knockdown of *raptor*, which encodes a component of mTORC1 [7,8,19] using a morpholino antisense oligo nucleotide (MO) (Additional file 4: Figure S4). Taken together, inhibition of mTORC1 signaling with these three inhibitors (rapamycin, Torin1, and AZD8055) or by knockdown of *raptor* suggests that mTORC1 signaling is required in the pre-blastema formation, blastema formation, and regenerative outgrowth stages during fin regeneration.

**Figure 4:**
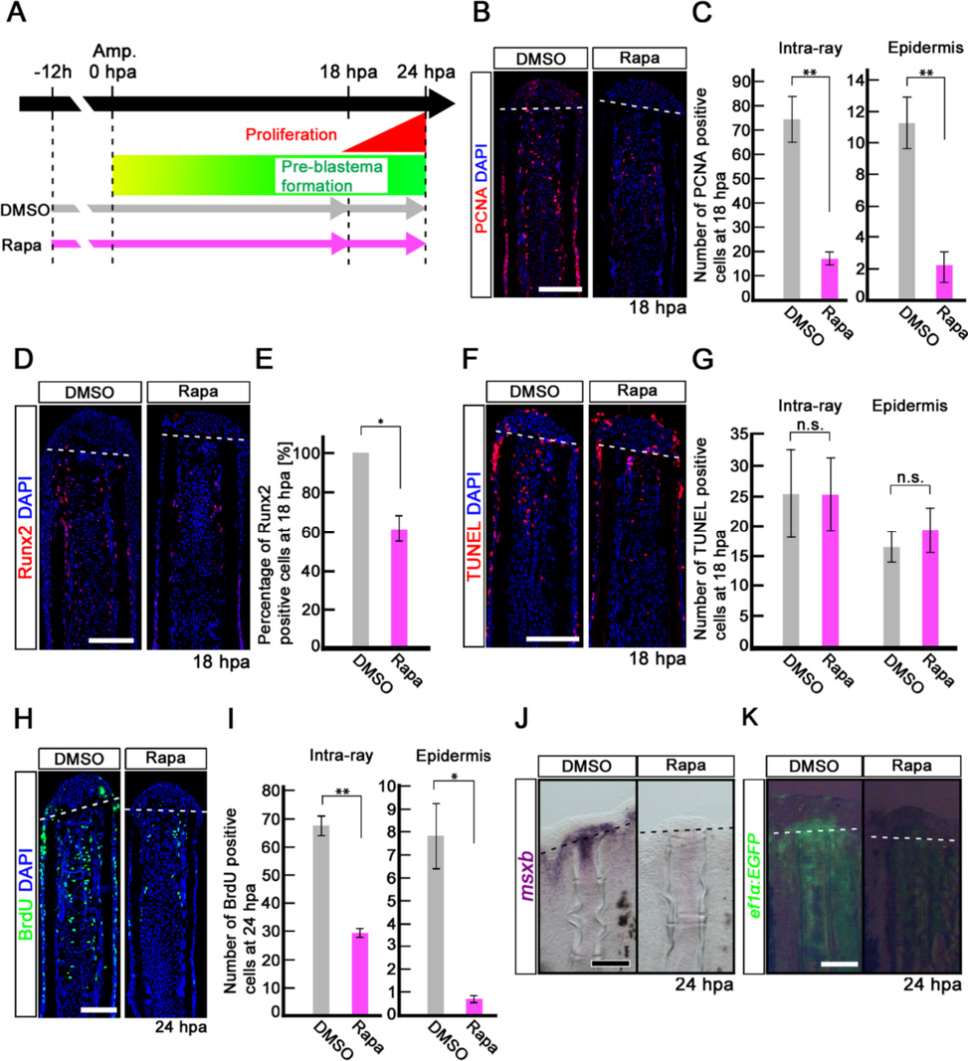
**Rapamycin treatment inhibits proliferation of intra-ray and epidermal cells, but not apoptosis before blastema formation. (A)** Scheme of rapamycin treatment before blastema formation. **(B, C)** PCNA-stained fin sections and quantification of PCNA-positive cells in the intra-ray and epidermis at 18 hpa. The number of PCNA-positive cells was significantly reduced by rapamycin treatment in both the intra-ray and epidermis at 18 hpa. **p < 0.01 by Student’s *t*-test. Error bars represent the standard error of 5 independent experiments. Scale bars: 100 μm. **(D,E)** Runx2-stained fin sections and quantification of Runx2-positive cells in the intra-ray. Rapamycin treatment significantly reduced the percentage of Runx2-positive cells at 18 hpa. *p < 0.05 by Student’s *t*-test. Error bars represent the standard error of 3 independent experiments. Scale bars: 100 μm. **(F, G)** TUNEL-stained fin sections and quantification of TUNEL-positive intra-ray and epidermal cells. Cell death was not increased in both the intra-ray and epidermis at 18 hpa. Error bars represent the standard error of 6 independent experiments. Scale bars: 100 μm. **(H, I)** BrdU-stained fin sections and quantification of BrdU-positive cells in the intra-ray and epidermis. Rapamycin treatment significantly reduced the number of BrdU-positive cells in the intra-ray and epidermis at 24 hpa. *p < 0.05 , **p < 0.01 by Student’s *t*-test. Error bars represent the standard error of 3 independent experiments. Scale bars: 100 μm. DAPI fluorescent signal (blue) indicates the presence of nuclei **(B, D, F, H)**. Dashed white lines indicate the amputation planes **(B, D, F, H)**. **(J)** Expression of *msxb* was examined by *in situ* hybridization at 24 hpa (n = 3). The *msxb* expression was barely detectable in rapamycin-treated fin regenerates. Scale bars: 200 μm. **(K)** EGFP fluorescence of Tg(*ef1*-*α*;*EGFP*) fin regenerates at 24 hpa (n = 3). The EGFP fluorescence was lost in rapamycin-treated fin regenerates. Scale bars: 200 μm. Dashed lines indicate the amputation plane **(J, K)**.

We showed that mTORC1 signaling is active in proliferative intra-ray cells and osteoblast progenitors during the pre-blastema formation stage (Figure 2A-F’). To test whether mTORC1 signaling affects cell proliferation before blastema formation, PCNA and Runx2 immunohistochemical staining, a BrdU incorporation assay, and expression of *msxb* [20] and the *Xenopus ef1*-*α*:*EGFP* transgene using the transgenic fish XIG8A [Tg(*ef1*-*α*;*EGFP*)] [21] were performed in rapamycin-treated fins. Because cell proliferation starts at approximately 18 hpa in regenerating fins [3], inhibition experiments of mTORC1 signaling by rapamycin treatment were performed from -12 h to 18 hpa (or 24 hpa). mTORC1 signaling inhibition was confirmed by the loss of the p-S6K signal at 24 hpa (Additional file 5: Figure S5). At 18 hpa, the number of PCNA-positive cells and the percent of Runx2-positive cells were significantly reduced by rapamycin treatment (Figure 4B-E), whereas the number of apoptotic cells was not increased in the intra-ray and epidermal cells (Figure 4F,G), indicating that inhibition of mTORC1 signaling suppresses cell proliferation without inducing apoptosis. Consistent with PCNA and Runx2 immunohistochemical staining, the number of BrdU incorporated cells in both the intra-ray and epidermis was significantly reduced by the rapamycin treatment at 24 hpa (Figure 4H,I). It was previously reported that *msxb* and *Xenopus ef1*-*α*:*EGFP* transgene are molecular markers for mesenchymal progenitor cells [20] and proliferative cells [22] in the regenerating fins, respectively. Similarly to PCNA and Runx2 expression, *msxb* and *Xenopus ef1*-*α*:*EGFP* transgene expression was markedly decreased by rapamycin treatment at 24 hpa as determined by whole-mount *in situ* hybridization and EGFP fluorescence, respectively (Figure 4J,K). These results clearly indicate that mTORC1 signaling is required for cell proliferation, but not in cell survival of intra-ray and epidermal cells before blastema formation.

### mTORC1 signaling is required for cell proliferation and cell survival during the regenerative outgrowth stage

**Figure 5:**
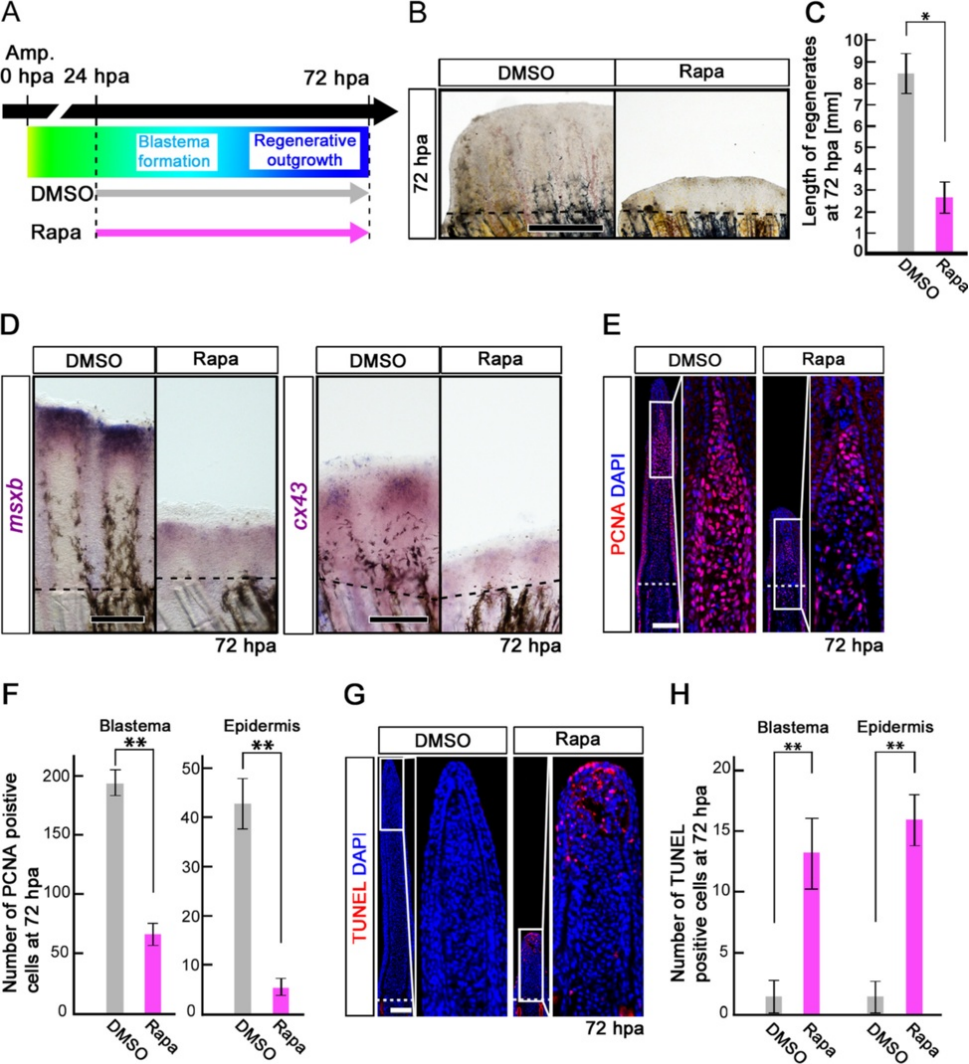
**Rapamycin treatment inhibits both the proliferation and survival of intra-ray cells during the blastema formation and regenerative outgrowth stages. (A)** Scheme of rapamycin treatment during blastema formation and regenerative outgrowth stages. **(B, C)** Rapamycin treatment significantly blocked the outgrowth of fin regenerates at 72 hpa. *p < 0.05 by Student’s *t*-test. Error bars represent the standard error of 4 independent experiments. Scale bars: 500 μm. Dashed lines indicate the amputation plane. **(D)** Expression of *msxb* and *cx43* was examined by whole-mount *in situ* hybridization at 72 hpa (*msxb*, n = 5; *cx43*, n = 3). Rapamycin treatment induced the down-regulation of *msxb* and *cx43* expression. Scale bars: 200 μm. Dashed lines indicate the amputation plane. **(E, F)** PCNA-stained fin sections and quantification of PCNA-positive cells in the intra-ray and epidermis at 72 hpa. The number of PCNA-positive cells was significantly reduced by rapamycin treatment in both the blastema and epidermis at 72 hpa. **p < 0.01 by Student’s *t*-test. Error bars represent the standard error of 4 independent experiments. Scale bars: 100 μm. **(G, H)** TUNEL-stained fin sections and quantification of TUNEL-positive cells in the intra-ray and epidermis. Cell death was significantly increased by rapamycin treatment in both the blastema and epidermis at 72 hpa. **p < 0.01 by Student’s *t*-test. Error bars represent the standard error of 4 independent experiments. Scale bars: 100 μm. DAPI fluorescent signal (blue) indicates the presence of nuclei **(E, G)**.

Because p-S6K-positive cells start to accumulate underneath the wound epidermis from 24 hpa (Figure 1E), and cell proliferation is suppressed until 24 hpa by mTORC1 signaling inhibition (Figure 4), identifying the function of mTORC1 signaling during blastema formation and regenerative outgrowth is difficult. We next examined the function of mTORC1 signaling during the blastema formation and regenerative outgrowth stages using rapamycin from 24 to 72 hpa (Figure 5A). Regenerative outgrowth was significantly inhibited by rapamycin treatment from 24 to 72 hpa (Figure 5B,C), as observed by rapamycin treatment from -12 h to 72 hpa (Figure 3). mTORC1 signaling inhibition was confirmed by the loss of the p-S6K signal at 72 hpa (Additional file 6: Figure S6). In addition, *msxb* and *connexin43* (*cx43*), a molecular marker of proliferating cells [23], expression was downregulated by rapamycin treatment in the regenerative fins (Figure 5D). Consistent with these *in situ* hybridization results, the number of PCNA-positive cells in both the blastema and epidermis was significantly reduced by rapamycin treatment (Figure 5E,F), as observed before blastema formation (Figure 4). In contrast to the pre-blastema formation stage, the number of apoptotic cells in both the blastema and epidermis was significantly increased by rapamycin treatment during the blastema formation and regenerative outgrowth stages (Figure 5G,H). These results suggest that mTORC1 signaling is required for cell proliferation and cell survival during blastema formation and regenerative outgrowth.

### mTORC1 signaling is required for the proliferation and differentiation of bony fin ray after 72 hpa

**Figure 6:**
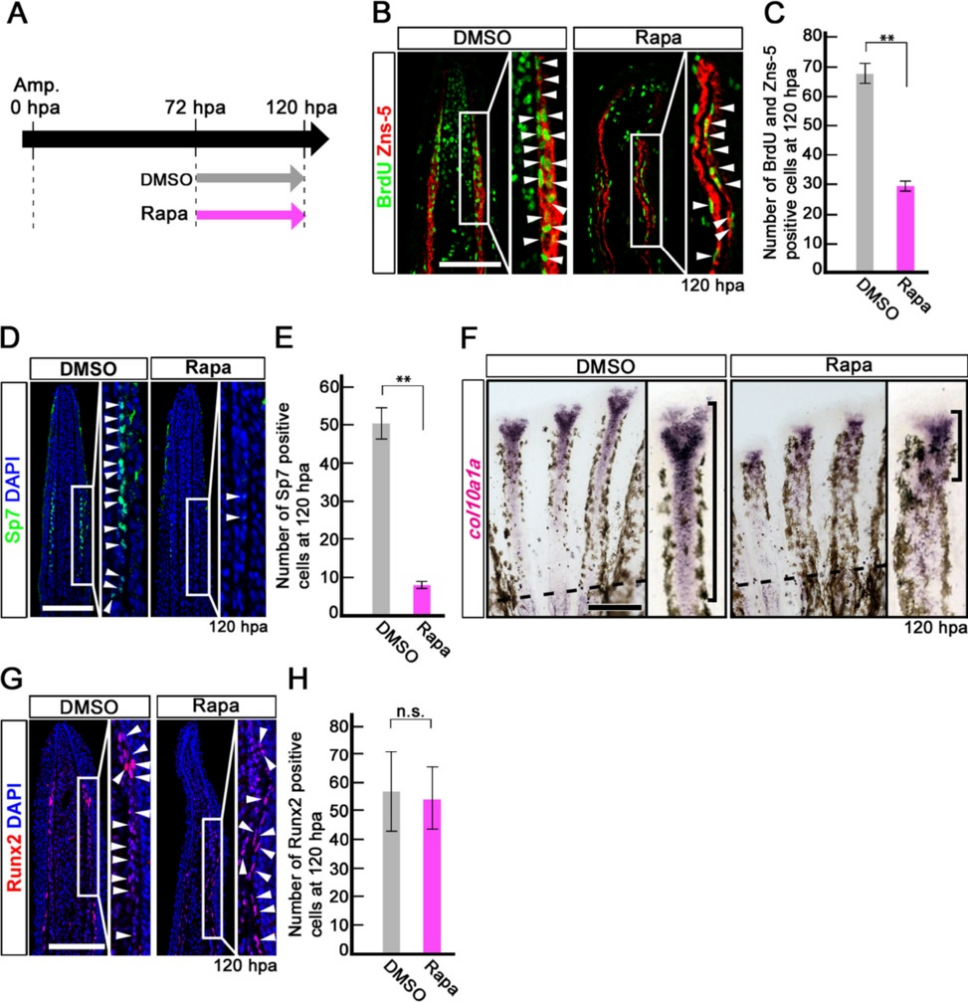
**Rapamycin treatment inhibits proliferation and differentiation of osteoblasts after 72 hpa. (A)** Scheme of rapamycin treatment between 72 and 120 hpa. **(B, C)** BrdU and Zns-5 double-stained fin sections and quantification of BrdU and Zns-5 double-positive osteoblasts. The number of double-positive osteoblasts was significantly reduced by rapamycin treatment at 120 hpa. **p < 0.01 by Student’s *t*-test. Error bars represent the standard error of 4 independent experiments. **(D, E)** Sp7-stained fin sections and quantification of Sp7-positive osteoblasts. The number of Sp7-positive osteoblasts was significantly reduced by rapamycin treatment at 120 hpa. DAPI fluorescent signal (blue) indicates the presence of nuclei. **p < 0.01 by Student’s *t*-test. Error bars represent the standard error of 4 independent experiments. Scale bars: 100 μm. **(F)** Expression of *col10a1a* was examined by *in situ* hybridization at 120 hpa (n = 4). Rapamycin treatment decreased *col10a1a* expression (brackets in F) at 120 hpa. Dashed lines indicate the amputation planes. Scale bars: 200 μm. **(G, H)** Runx2-stained fin sections and quantification of Runx2-positive cells. The number of Runx2-positive cells was not affected by rapamycin treatment at 120 hpa. Error bars represent the standard error of 4 independent experiments. Scale bars: 100 μm.

As shown in Figure 2, mTORC1 signaling was specifically activated in the putative differentiating osteoblasts after 72 hpa. To examine the function of mTORC1 signaling in bony ray formation, the regenerates were treated with rapamycin from 72 to 120 hpa. Inhibition of mTORC1 signaling was confirmed by the marked reduction of the p-S6K signal at 120 hpa (Additional file 7: Figure S7). The number of BrdU incorporated cells in the Zns-5-positive osteoblasts was significantly reduced by rapamycin treatment (Figure 6B,C). In addition, the number of Sp7-expressing cells and expression domains of *col10a1a*, which are intermediate markers of skeletogenesis [15], were markedly decreased in the rapamycin-treated fins (Figure 6D,E,F). In contrast to differentiation markers for skeletogenesis, the number of Runx2-positive osteoblast progenitors was unchanged by rapamycin treatment (Figure 6G,H). These results suggest that mTORC1 signaling is required for proliferation and differentiation of the bony fin ray after 72 hpa.

### mTORC1 signaling does not regulate autophagy in fin regeneration

A recent study revealed that autophagy is required for zebrafish fin regeneration under the control of MAPK/Erk signaling pathway [24]. Because the mTORC1 signaling pathway is known to inhibit autophagy [7,8], we examined whether autophagy was affected by inhibition of mTORC1 signaling during fin regeneration. As determined using a GFP-microtubule-associated protein 1 light chain 3 isoform (GFP-LC3) transgenic line, autophagy was markedly upregulated from 1 to 4 days post amputation (dpa) [24]. Using an LC3B antibody, we detected LC3 in the wound epidermis at 24 hpa, and by 72 hpa, LC3 localization in the wound epidermis was maintained (Additional file 8: Figure S8). Moreover, LC3 protein level and localization were not affected by rapamycin treatment (Additional file 8: Figure S8), suggesting that the mTORC1 signaling pathway does not regulate fin regeneration via autophagy.

### IGF-1 receptor (IGF-1R)/PI3K and Wnt signaling pathways regulate mTORC1/S6K in fin regeneration

**Figure 7:**
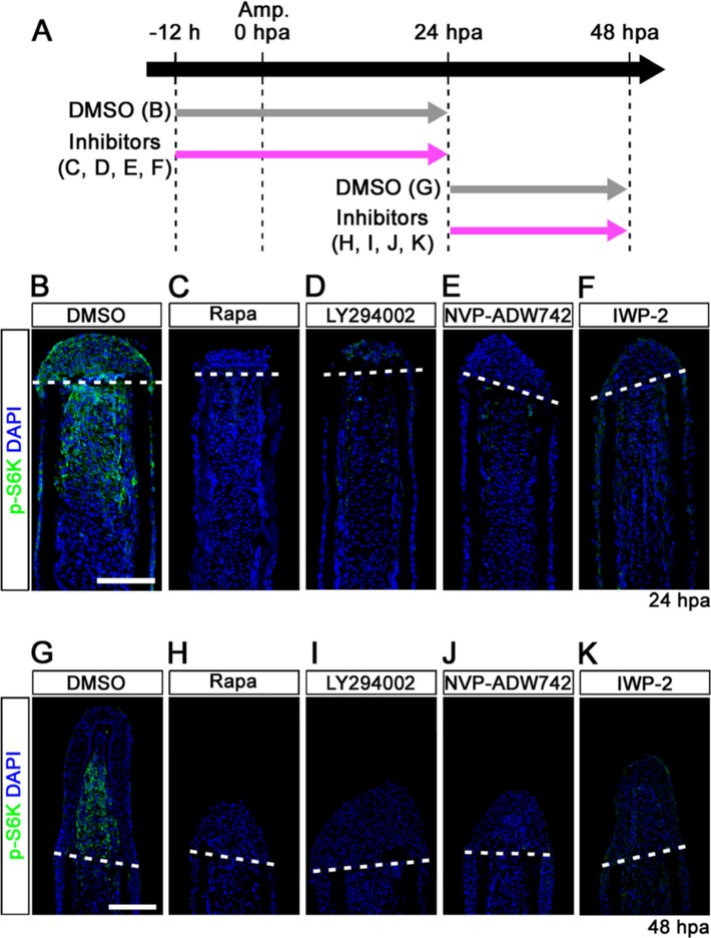
**IGF-1R/PI3K and Wnt pathways regulate the activation of mTORC1 during fin regeneration. (A)** Scheme of inhibitor treatments of rapamycin, LY294002 (a PI3K inhibitor), NVP-ADW742 (an IGF-1R inhibitor), or IWP-2 (a Wnt/β-catenin inhibitor) during fin regeneration. **(B-K)** Longitudinal sections of DMSO or inhibitors treated wild-type fin regenerates that were immunohistochemically stained with an antibody against p-S6K (green) at 24 (DMSO, n = 6; Rapa, n = 4; LY294002, n = 4; NVP-ADW742, n = 4; IWP-2, n = 5) and 48 hpa (DMSO, n = 6; Rapa, n = 4; LY294002, n = 4; NVP-ADW742, n = 4; IWP-2, n = 4). DAPI fluorescent signal (blue) indicates the presence of nuclei. The activation of S6K was blocked by LY294002, NVP-ADW742, or IWP-2 treatment. Dashed white lines indicate the amputation planes. Scale bars: 100 μm.

It is well known that the mTORC1/S6K pathway is regulated through the receptor tyrosine kinase/PI3K/Akt pathway in cell proliferation and metabolism [7,8]. To examine the upstream signaling pathway regulating mTORC1/S6K signaling in fin regeneration, various inhibitors for IGF, Wnt, Fgf, and ROS signaling pathways were tested from -12 h to 24 hpa or from 24 to 48 hpa, and S6K activation was determined; IGF signaling (LY294002 [25]: a PI3K inhibitor, NVP-ADW742 [26]: a IGF-1 receptor kinase inhibitor), Wnt signaling (IWP-2 [27]: a Wnt/β-catenin signaling inhibitor), Fgf signaling (SU5402 [28]: a Fgf receptor1 inhibitor), MAPK/Erk signaling (U0126 [29]: a MAPK/Erk inhibitor), and ROS signaling (VAS2870 [30]: an inhibitor of NADPH oxidase). No inhibitory effect for S6K activation was observed using SU5402, U0126, or VAS2870, even though fin regeneration was suppressed (Additional file 9: Figure S9). However, LY294002, NVP-ADW742, and IWP-2 inhibitor treatment markedly reduced the p-S6K signal in blastema and epidermal cells. The inhibitory effect of LY294002, NVP-ADW742, and IWP-2 on S6K activation was nearly similar to that of rapamycin during fin regeneration (Figure 7). These results suggest that both IGF-1R/PI3K and Wnt pathways activate mTORC1 in blastema and epidermal cells during zebrafish caudal fin regeneration.

## Discussion

Based on the localization of p-S6K, we show the spatiotemporal distributions of cells in which mTORC1 signaling is activated during fin regeneration (Figure 1). Weak p-S6K fluorescent signals were observed in the intra-ray cells and epidermis as early as 6 hpa. Further, the p-S6K-positive cells were found to be distributed in locations proximal to the amputation plane by 36 hpa, indicating that mTORC1 is one of the earliest signaling in fin regeneration, which is reminiscent of other signaling pathways such as Wnt, Fgf, retinoic acid, and Activin [2,9,10]. We also show that mTORC1 signaling is active in proliferative blastema cells, wound epidermal cells, and osteoblasts during blastema formation and regenerative outgrowth (after 24 hpa) (Figure 2). Inhibition of mTORC1 signaling from 24 to 72 hpa suppresses proliferation of these cells through the inhibition of S6K activation (Figure 5, Additional file 6: Figure S6). These results suggest that mTORC1 signaling is continuously required for cellular proliferation during the blastema formation and regenerative outgrowth stages via protein synthesis.

It is well established that mTORC1 signaling activates anabolic processes, including the synthesis of proteins, nucleotides, and lipids, and, as a result, these processes promote cell growth and proliferation [7,8,31]. Protein and nucleotide syntheses are controlled through the activation of S6K, and lipid synthesis is promoted by activating sterol regulatory element binding proteins in an S6K-dependent or -independent manner [8,31]. Based on these findings, the p-S6K-positive cells observed in regenerating fins appear to be in a higher metabolic state that is stimulated by a wound signal. It would be significant to isolate and analyze these cells under high metabolic conditions during fin regeneration.

A recent paper demonstrated that zebrafish caudal fin regeneration is slightly inhibited by rapamycin treatment from 0 to 35 dpa [32]. However, the inhibitory effect of rapamycin observed was milder than the effect observed in our study. The rapamycin concentration used in this report was lower than that used in the present study (50 nM in a previous report versus 2.4 μM in the present study), which may explain this discrepancy. In addition, while fish were pretreated with rapamycin before amputation (-12 h) in our experiments, no pretreatment was performed in the previous report. In fact, we found that at least 6 h of pretreatment is needed to observe the loss of p-S6K immunostaining. When fish were treated with rapamycin from 0 hpa, the inhibitory effect of rapamycin on fin regeneration was markedly reduced, even when using a 2.4 μM concentration of rapamycin (data not shown). It is likely that these two different experimental conditions may be responsible for the difference observed between our study and the previous report.

A previous report showed that *insulin*-*like growth factor 2b* (*igf2b*), which is expressed in blastema, activates the IGF signaling pathway in the apical epithelium cells through two receptors, insulin-like growth factor 1a receptor (Igf1ra) and Igf1rb [33]. Pharmacological inhibition using NVP-ADW541 or NVP-ADW742 caused apoptosis in the wound epidermis and blocked blastema formation, indicating that IGF signaling between blastema and wound epidermis is critical for cell survival in the wound epidermis and cell proliferation in the blastema [33]. These results are consistent with some of our results showing that mTORC1 signaling is active in the wound epidermis before blastema formation, and is regulated by the IGF-1R/PI3K signaling pathway. However, two phenotypic differences were observed between mTORC1 and IGF-1R inhibition during fin regeneration. One is that cell death was significantly increased in the wound epidermis by IGF-1R inhibition at 24 hpa [33], but not by rapamycin treatment from -12 h to 18 hpa (Figure 4). Because the IGF signaling is one of upstream signaling leading to mTORC1/S6K pathway, the other signaling pathways might be involved in the wound epidermis survival. The second is that activation of mTORC1 based on p-S6K localization was observed not only in the wound epidermis, but also in intra-ray cells as early as 6 hpa. It is possible that, in intra-ray cells, mTORC1 signaling is activated through IGF-1Rs, other than Igf1ra and Igf1rb.

In addition to IGF-1R/PI3K signaling, we also found that Wnt signaling controls mTORC1 activation in both blastema and epidermal cells during fin regeneration by using IWP-2 (Figure 7). It has already been shown that Wnt signaling directly activates mTORC1 through the inhibition of glycogen synthase kinase 3 and tuberous sclerosis 1/2 in zebrafish and mice [34], as well as in mammalian cell lines [35]. In zebrafish caudal fin regeneration, Wnt/β-catenin signaling indirectly regulates the proliferation of blastema cells, patterning of epidermis, and differentiation and patterning of bone, mediated through many signaling pathways such as Activin, Notch, Fgf, retinoic acid, Hedgehog, IGF, and Bmp [10]. Because mTORC1 signaling could be mediated directly or indirectly through IGF signaling regulated by Wnt/β-catenin signaling, further detailed investigations are needed to elucidate the hierarchical relationship and crosstalk between IGF-1R/PI3K/mTORC1 and other signaling pathways downstream of Wnt/β-catenin during fin regeneration.

## Conclusions

In this study, we showed that the mTORC1 signaling is activated in intra-ray and wound epidermal cells as early as 6 hpa, and continues to be activated in proliferative blastema cells, wound epidermal cells, and osteoblasts during fin regeneration. Inhibition of mTORC1 signaling during the pre-blastema formation, blastema formation, and regenerative outgrowth stages by rapamycin suggests that mTORC1 signaling is required for fin regeneration. Before blastema formation, mTORC1 signaling is required for intra-ray and wound epidermal cell proliferation, but not in cell survival, whereas during the blastema formation and regenerative outgrowth stages, mTORC1 signaling is required for both blastema and wound epidermal cell proliferation and survival. Furthermore, during the regenerative outgrowth stage, mTORC1 signaling is required for both proliferation and differentiation of the bony fin ray. In summary, our study is the first report that uncovers the requirement and significance of mTORC1 signaling for the cell proliferation, cell survival, or differentiation of intra-ray cells, blastema cells, wound epidermal cells, and/or osteoblasts during vertebrate epimorphic regeneration downstream of IGF and Wnt signaling pathways.

## Methods

### Zebrafish husbandry, fin amputation, drug treatments, and *in vivo* electroporation

All zebrafish experiments were performed under ethical approval of the Hiroshima University Animal Research Committee (Permit Number: F13-1). Maintenance and caudal fin amputation of adult zebrafish (AB/Tüebingen strain) were performed as described previously [36]. Transgenic zebrafish XIG8A [Tg(*ef1*-*α*:*EGFP*)] [21] was used for fin amputation experiments.

During drug treatments, fish were kept in fish water at 28.5°C and the fish water with drug was replaced daily. 2.4 μM rapamycin (LC Laboratories), 100 nM Torin1 (Calbiochem), 1.2 μM AZD8055 (AdooQ Biosciences), 10 μM LY294002 (Calbiochem), 5 μM NVP-ADW742 (AdooQ Biosciences), 14 μM SU5402 (Calbiochem), 10 μM IWP-2 (Promega), 25 μM U0126 (Promega), and 1 μM VAS2870 (Enzo Life Sciences) were used as specific inhibitors. All were dissolved in DMSO and final DMSO concentration in fish water was 0.1%, except SU5402 (0.17%). The control fish were kept in fish water with 0.1% DMSO.

For MO knockdown experiments, *in vivo* electroporation was performed as described previously [37]. The MO was micro-injected between each bony fin ray and electroporated before fin amputation. We used fluorescent tagged-MO targeted against *raptor* and 5-base mismatch control MO (Gene Tools, Inc.) as following; a raptor MO (5′-ATGGATGGATGGATGCTCACCTATC-3′) [19], a 5-mismatch control MO (5′- ATGaATGaATGaATGaTCACaTATC -3′, lower case letters indicate mismatch pairs).

### Immunohistochemical staining and whole-mount *in situ* hybridization

The amputated fins were fixed in 4% paraformaldehyde in 0.1 M phosphate-buffered saline (PBS) overnight at 4°C. After fixation, the fins were treated with PBS containing 30% sucrose overnight, and were embedded with Tissue-Tek O.C.T. compound (Sakura Finetek). The embedded fins were frozen and sectioned to 14 μm thickness by using a Leica CM3050S. The sections were dehydrated and rehydrated through a methanol/PBS-0.1% Tween series for 5 min each. After rehydration, the sections were blocked with PBST (PBS and 0.1% Tween20) containing 5% sheep serum for 3 h at room temperature and then incubated in PBST with primary antibody/antibodies overnight at 4°C. The following primary antibodies were used: anti-PCNA mouse monoclonal antibody at 1:1000 (Sigma, #P8825) [36]; anti-BrdU rat monoclonal antibody at 1:100 (Abcam, ab6326); phospho-S6 ribosomal protein (Ser240/244) rabbit polyclonal antibody at 1:300 (Cell Signaling, #2215) [38]; anti-Runx2 mouse monoclonal antibody at 1:100 (Sant Cruz Biotechnology, 27-K) [10,11]; anti-Zns-5 mouse monoclonal antibody at 1:300 (a kindly gift from Dr. Ishitani) [14]; anti-Sp7 rabbit polyclonal antibody at 1:1000 (Santa cruz, sc-22536-R) [11]; anti-LC3B rabbit polyclonal antibody at 1:300 (Abcam, ab51520) [39]. The following secondary antibodies were used: Alexa Fluor® 488 goat anti-rabbit IgG antibody at 1:500 (Invitrogen, Life Technologies Corp.); Alexa Fluor® 488 goat anti-rat IgG antibody at 1:500 (Invitrogen, Life Technologies Corp.); Alexa Fluor® 594 goat anti-mouse IgG antibody at 1:500 (Invitrogen, Life Technologies Corp.); Alexa Fluor® 594 goat anti-rabbit IgG Antibody at 1:500 (Invitrogen, Life Technologies Corp.). 4′,6-diamidino-2-phenylindole (DAPI) was used for nuclei staining at a concentration of 1:1000. The images were captured using an Olympus FV1000-D confocal microscope with the same exposure times using the FluoView software.

Whole-mount *in situ* hybridization analyses were performed as described previously [40] with 60 minute proteinase K treatment.

### BrdU incorporation assays and cell death detection

BrdU incorporation assays were performed as described previously [36]. Fin-amputated fish were allowed to regenerate in the fish water containing with 50 μg/ml BrdU between 18 to 24 hpa or 108 to 120 hpa. After incubation, the regenerating fins were cut and BrdU-labeled cells were detected as described previously [36]. For detection of apoptotic cells, we performed TUNEL staining using an *In Situ* Cell Death Detection Kit (Roche Applied Science) according to the manufacture’s instruction.

## Additional files

Additional file 1: Figure S1. Treatment of Torin1 or AZD8055 inhibits fin regeneration until 72 hpa. (A) Scheme of Torin1 or AZD8055 treatment from – 12 h to 72 hpa. (B,C) Treatment of both inhibitors significantly inhibited fin regeneration from – 12 h to 72 hpa (pre-blastema formation, blastema formation, and regenerative outgrowth stages), when compared to DMSO treatment. Dashed lines indicate the amputation planes. **p* < 0.05 , ***p* < 0.01 by Student’s *t*-test. Error bars represent the standard error of 4 independent experiments. Scale bars: 500 μm in B. Additional file 2: Figure S2. Distributions of p-S6K in rapamycin, Torin1, or AZD8055-treated fin regenerates at 72 hpa. (A) Scheme of rapamycin, Torin1, or AZD8055 treatment from – 12 h to 72 hpa. (B) Longitudinal sections of wild-type fin regenerates that were immunohistochemically stained with an antibody against p-S6K (green) at 72 hpa (DMSO, n = 5; Rapa, n = 3; Torin1, n = 3; AZD8055, n = 3). DAPI fluorescent signal (blue) indicates the presence of nuclei. At 72 hpa, the p-S6K fluorescent signal was lost in rapamycin, Torin1, or AZD8055-treated fin regenerates. Dashed white lines indicate the amputation plane. Scale bars: 100 μm. Additional file 3: Figure S3. p-S6K signals were markedly reduced by 3 or 6 h treatment with inhibitors (rapamycin, Torin1, and AZD8055). (A) Scheme of rapamycin, Torin1, or AZD8055 treatment from 24 h to 27 or 30 hpa. (B-I) Longitudinal sections of wild-type fin regenerates that were immunohistochemically stained with an antibody against p-S6K (green) at 27 hpa (DMSO, n = 3; Rapa, n = 3; Torin1, n = 3; AZD8055, n = 3) or 30 hpa (DMSO, n = 3; Rapa, n = 3; Torin1, n = 3; AZD8055, n = 3). DAPI fluorescent signal (blue) indicates the presence of nuclei. The p-S6K signals were markedly reduced by 3 or 6 h treatment with inhibitors (rapamycin, Torin1, and AZD8055). Dashed white lines indicate the amputation plane. Scale bars: 100 μm. Additional file 4: Figure S4. Fin regeneration and activation of mTORC1 signaling are inhibited by *raptor* knock-down. (A,B) Wild-type fin electroporated in the ventral half with fluorescein-labeled MOs at 72 hpa. Fin regeneration was significantly inhibited by *raptor* knock-down. Dashed lines indicate the amputation planes. Scale bars: 500 μm. ***p* < 0.01 by Student’s *t*-test. Error bars represent the standard error of 5 independent experiments. (C) Longitudinal sections of wild-type fin regenerates that were immunostained with antibodies against p-S6K (red) at 24 hpa (5-mismached *raptor* MO, n = 5; *raptor* MO, n = 5). Green fluorescence indicates the presence of MO in the electroporated cells. Merged views showed that cells incorporated 5-mismached *raptor* MO, but not *raptor* MO, are p-S6K-positive (arrowheads). It should be noted that fluorescent signals of 5-mismached *raptor* MO and p-S6K overlap only in the cytosol, because p-S6K are localized in the cytosol, not in the nucleus. Dashed white lines indicate the amputation planes. Scale bars: 100 μm. Additional file 5: Figure S5. Distributions of p-S6K in rapamycin-treated fin regenerates at 24 hpa. (A) Scheme of rapamycin treatment from – 12 h to 24 hpa. (B) Longitudinal sections of wild-type fin regenerates that were immunohistochemically stained with an antibody against p-S6K (green) at 24 hpa (DMSO, n = 4; Rapa, n = 4). DAPI fluorescent signal (blue) indicates the presence of nuclei. At 24 hpa, the p-S6K fluorescent signal was lost in rapamycin-treated fin regenerates. Dashed white lines indicate the amputation plane. Scale bars: 100 μm. Additional file 6: Figure S6. Distributions of p-S6K in fin regenerates treated with rapamycin from 24 to 72 hpa. (A) Scheme of rapamycin treatment from 24 to 72 hpa. (B) Longitudinal sections of wild-type fin regenerates that were immunohistochemically stained with an antibody against p-S6K (green) at 72 hpa (DMSO, n = 5; Rapa, n = 5). DAPI fluorescent signal (blue) indicates the presence of nuclei. At 72 hpa, the p-S6K fluorescent signal was lost in rapamycin-treated fin regenerates. Dashed white lines indicate the amputation plane. Scale bars: 100 μm. Additional file 7: Figure S7. Distributions of p-S6K in fin regenerates treated with rapamycin from 72 to 120 hpa. (A) Scheme of rapamycin treatment from 72 to 120 hpa. (B) Longitudinal sections of wild-type fin regenerates that were immunohistochemically stained with an antibody against p-S6K (green) at 120 hpa (DMSO, n = 3; Rapa, n = 3). DAPI fluorescent signal (blue) indicates the presence of nuclei. At 120 hpa, the fluorescent signal of p-S6K was markedly reduced by rapamycin treatment. Scale bars: 100 μm. Additional file 8: Figure S8. Distributions of LC3B in rapamycin treated fin regenerates. (A) Scheme of rapamycin treatment from -12 to 24 hpa or from 36 to 72 hpa. (B,C) Longitudinal sections of DMSO or rapamycin treated wild-type fin regenerates that were immunohistochemically stained with an antibody against LC3B (green) at 24 hpa (DMSO, n = 4; Rapa, n = 4) and 72 hpa (DMSO, n = 3; Rapa, n = 3). DAPI fluorescent signal (blue) indicates the presence of nuclei. LC3B is specifically localized in the wound epidermis at 24 and 72 hpa. Dashed white lines indicate the amputation planes. Scale bars: 100 μm. Additional file 9: Figure S9. Fgf, MAPK/Erk, and ROS signaling pathways do not regulate the activation of mTORC1 during fin regeneration. (A) Scheme of inhibitor treatments of SU5402 (a Fgf receptor1 inhibitor), U0126 (a MAPK/Erk inhibitor), and VAS2870 (an NADPH oxidase inhibitor: ROS signaling) during fin regeneration. (B-I) Longitudinal sections of DMSO or inhibitors treated wild-type fin regenerates that were immunohistochemically stained with an antibody against p-S6K (green) at 24 or 48 hpa. DAPI fluorescent signal (blue) indicates the presence of nuclei. The p-S6K signals in SU5402-, U0126-, and VAS2870-treated fins were approximately comparable to those in DMSO-treated fins at both 24 hpa (DMSO, n = 6; SU5402, n = 5; U0126, n = 3; VAS2870, n = 3) and 48 hpa (DMSO, n = 6; SU5402, n = 4; U0126, n = 3; VAS2870, n = 3), even though these inhibitors, at 48 hpa, prevented fin regeneration. Dashed white lines indicate the amputation planes. Scale bars: 100 μm.

## Abbreviations

BrdU: 5-bromo-2′-deoxyuridine
DAPI: 4′,6-diamidino-2-phenylindole
DMSO: Dimethyl sulfoxide
EGFP: Enhanced green fluorescent protein
qRT-PCR: quantitative real-time polymerase chain reaction
