# Supplementary figures and images for: Mechanistic target of rapamycin complex 1 signaling regulates cell proliferation, cell survival, and differentiation in regenerating zebrafish fins
Source: BMC Dev Biol. 2014 Dec 6;14:42. doi: 10.1186/s12861-014-0042-9 (PMC4264545; doi:10.1186/s12861-014-0042-9)

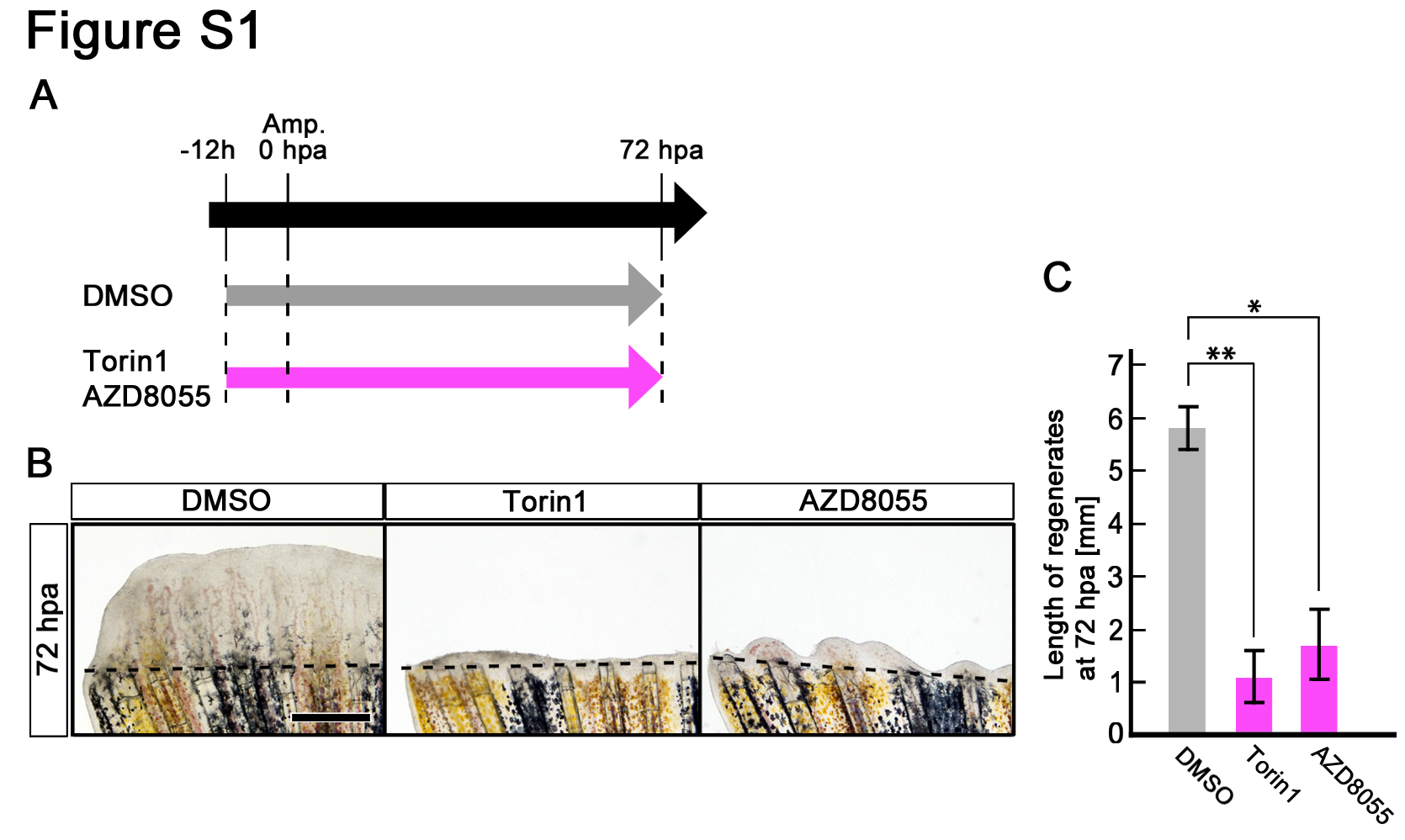

Supplement: Additional file 1: Figure S1. — Treatment of Torin1 or AZD8055 inhibits fin regeneration until 72 hpa. (A) Scheme of Torin1 or AZD8055 treatment from – 12 h to 72 hpa. (B,C) Treatment of both inhibitors significantly inhibited fin regeneration from – 12 h to 72 hpa (pre-blastema formation, blastema formation, and regenerative outgrowth stages), when compared to DMSO treatment. Dashed lines indicate the amputation planes. *p < 0.05 , **p < 0.01 by Student’s t-test. Error bars represent the standard error of 4 independent experiments. Scale bars: 500 μm in B. [file 12861_2014_42_MOESM1_ESM.tiff]

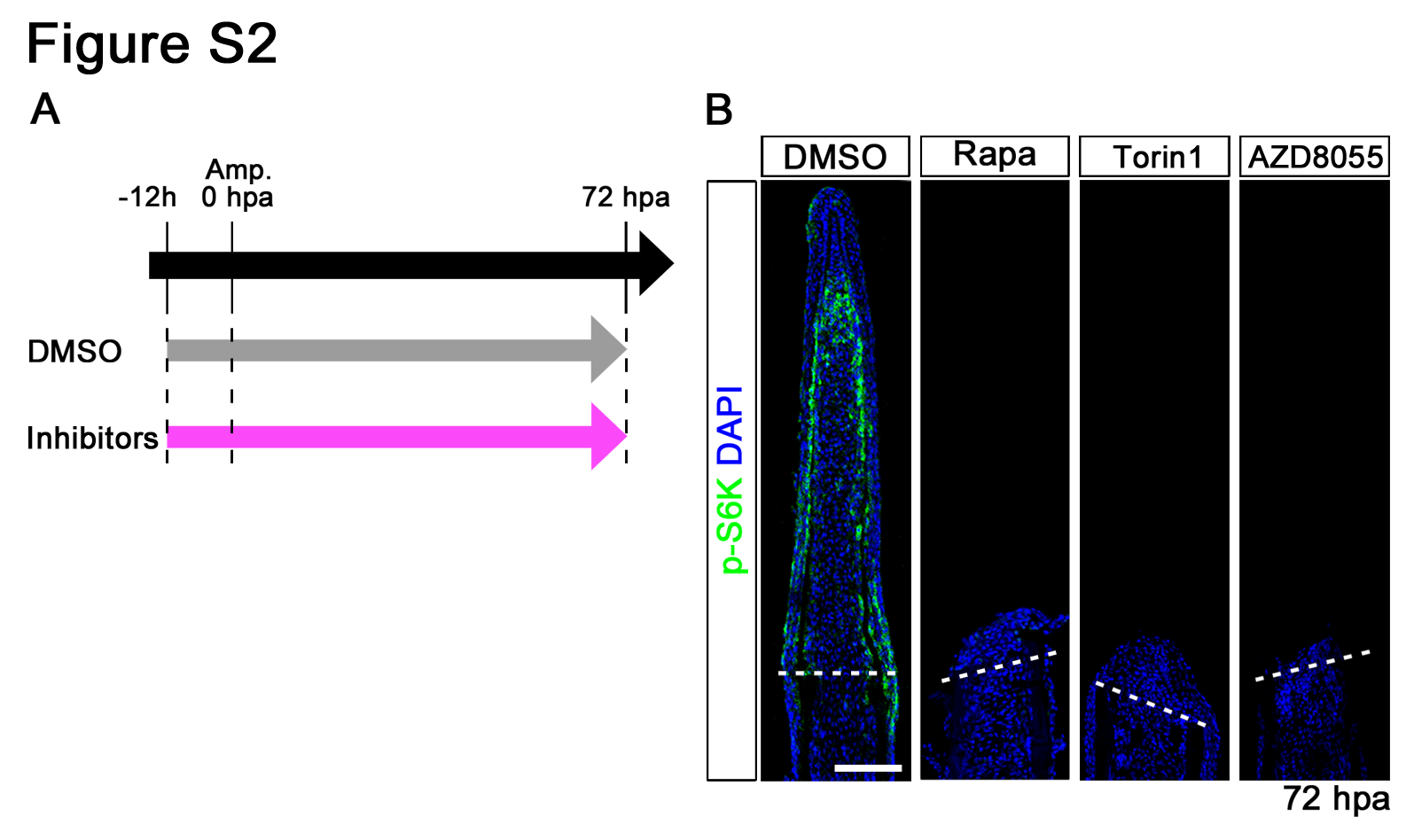

Supplement: Additional file 2: Figure S2. — Distributions of p-S6K in rapamycin, Torin1, or AZD8055-treated fin regenerates at 72 hpa. (A) Scheme of rapamycin, Torin1, or AZD8055 treatment from – 12 h to 72 hpa. (B) Longitudinal sections of wild-type fin regenerates that were immunohistochemically stained with an antibody against p-S6K (green) at 72 hpa (DMSO, n = 5; Rapa, n = 3; Torin1, n = 3; AZD8055, n = 3). DAPI fluorescent signal (blue) indicates the presence of nuclei. At 72 hpa, the p-S6K fluorescent signal was lost in rapamycin, Torin1, or AZD8055-treated fin regenerates. Dashed white lines indicate the amputation plane. Scale bars: 100 μm. [file 12861_2014_42_MOESM2_ESM.tiff]

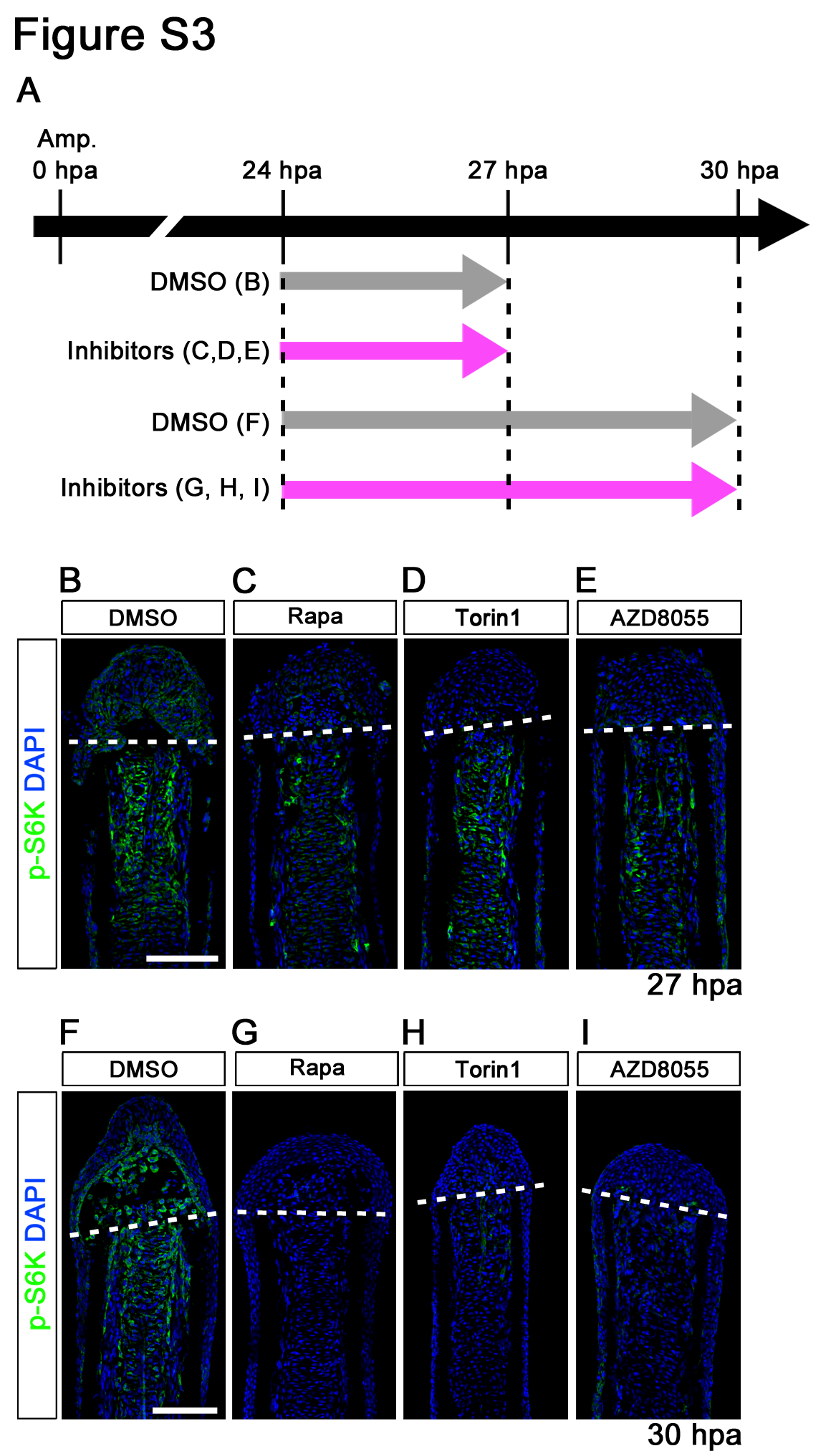

Supplement: Additional file 3: Figure S3. — p-S6K signals were markedly reduced by 3 or 6 h treatment with inhibitors (rapamycin, Torin1, and AZD8055). (A) Scheme of rapamycin, Torin1, or AZD8055 treatment from 24 h to 27 or 30 hpa. (B-I) Longitudinal sections of wild-type fin regenerates that were immunohistochemically stained with an antibody against p-S6K (green) at 27 hpa (DMSO, n = 3; Rapa, n = 3; Torin1, n = 3; AZD8055, n = 3) or 30 hpa (DMSO, n = 3; Rapa, n = 3; Torin1, n = 3; AZD8055, n = 3). DAPI fluorescent signal (blue) indicates the presence of nuclei. The p-S6K signals were markedly reduced by 3 or 6 h treatment with inhibitors (rapamycin, Torin1, and AZD8055). Dashed white lines indicate the amputation plane. Scale bars: 100 μm. [file 12861_2014_42_MOESM3_ESM.tiff]

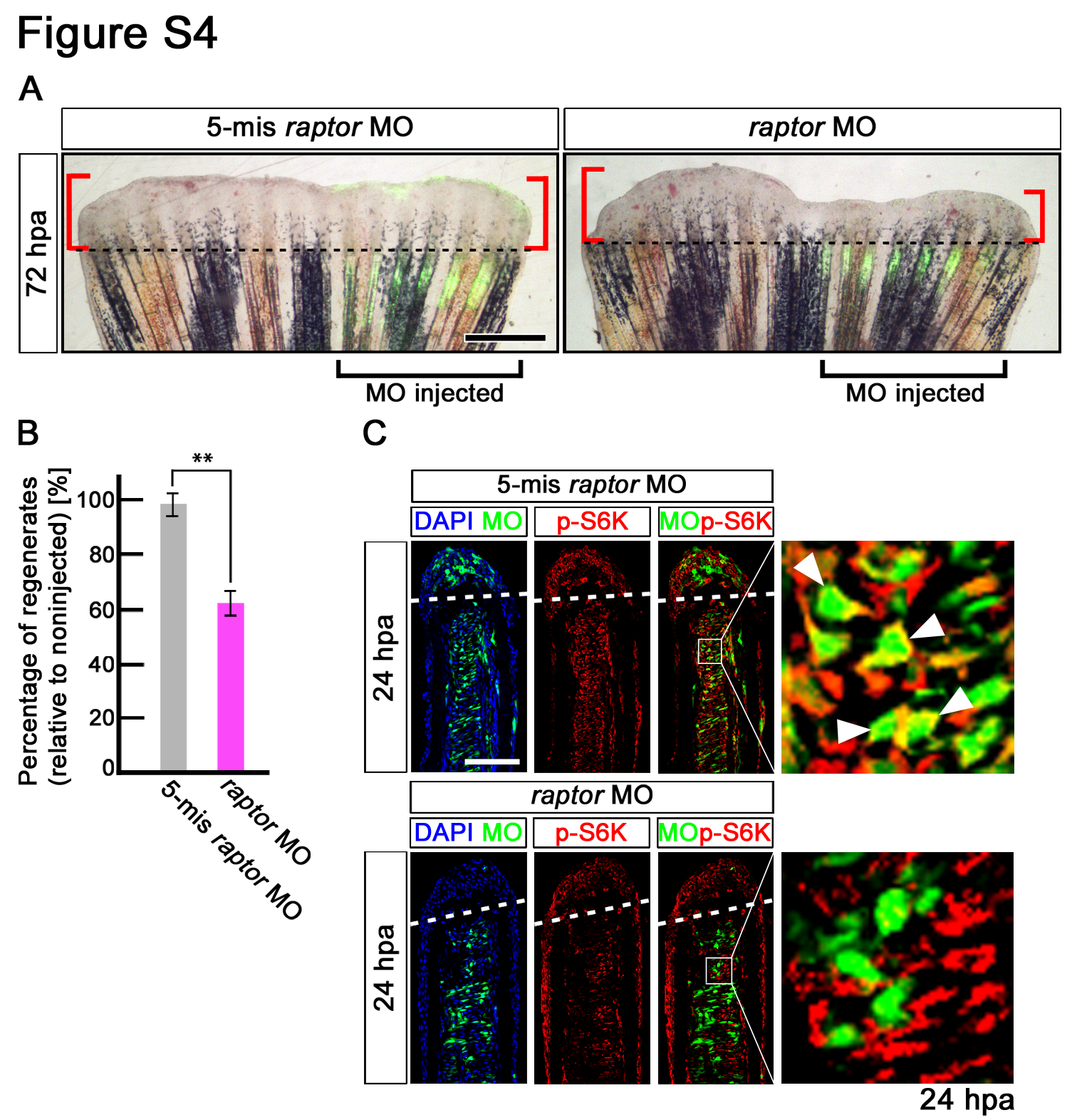

Supplement: Additional file 4: Figure S4. — Fin regeneration and activation of mTORC1 signaling are inhibited by raptor knock-down. (A,B) Wild-type fin electroporated in the ventral half with fluorescein-labeled MOs at 72 hpa. Fin regeneration was significantly inhibited by raptor knock-down. Dashed lines indicate the amputation planes. Scale bars: 500 μm. **p < 0.01 by Student’s t-test. Error bars represent the standard error of 5 independent experiments. (C) Longitudinal sections of wild-type fin regenerates that were immunostained with antibodies against p-S6K (red) at 24 hpa (5-mismached raptor MO, n = 5; raptor MO, n = 5). Green fluorescence indicates the presence of MO in the electroporated cells. Merged views showed that cells incorporated 5-mismached raptor MO, but not raptor MO, are p-S6K-positive (arrowheads). It should be noted that fluorescent signals of 5-mismached raptor MO and p-S6K overlap only in the cytosol, because p-S6K are localized in the cytosol, not in the nucleus. Dashed white lines indicate the amputation planes. Scale bars: 100 μm. [file 12861_2014_42_MOESM4_ESM.tiff]

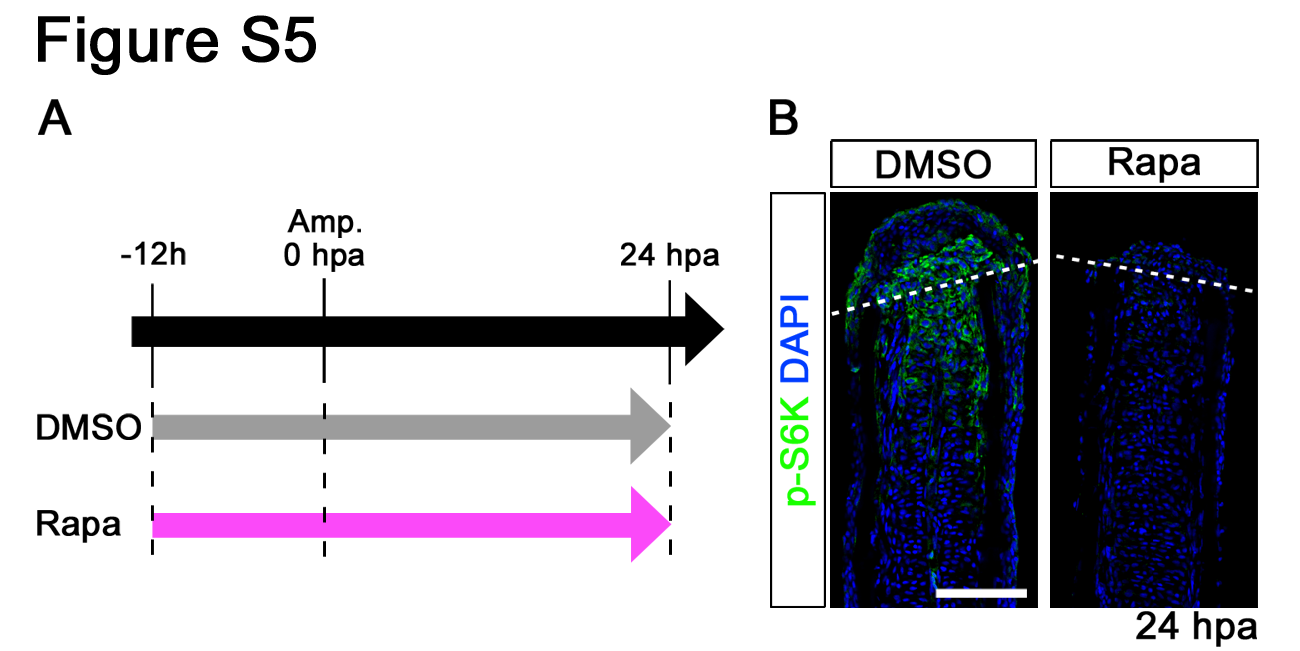

Supplement: Additional file 5: Figure S5. — Distributions of p-S6K in rapamycin-treated fin regenerates at 24 hpa. (A) Scheme of rapamycin treatment from – 12 h to 24 hpa. (B) Longitudinal sections of wild-type fin regenerates that were immunohistochemically stained with an antibody against p-S6K (green) at 24 hpa (DMSO, n = 4; Rapa, n = 4). DAPI fluorescent signal (blue) indicates the presence of nuclei. At 24 hpa, the p-S6K fluorescent signal was lost in rapamycin-treated fin regenerates. Dashed white lines indicate the amputation plane. Scale bars: 100 μm. [file 12861_2014_42_MOESM5_ESM.tiff]

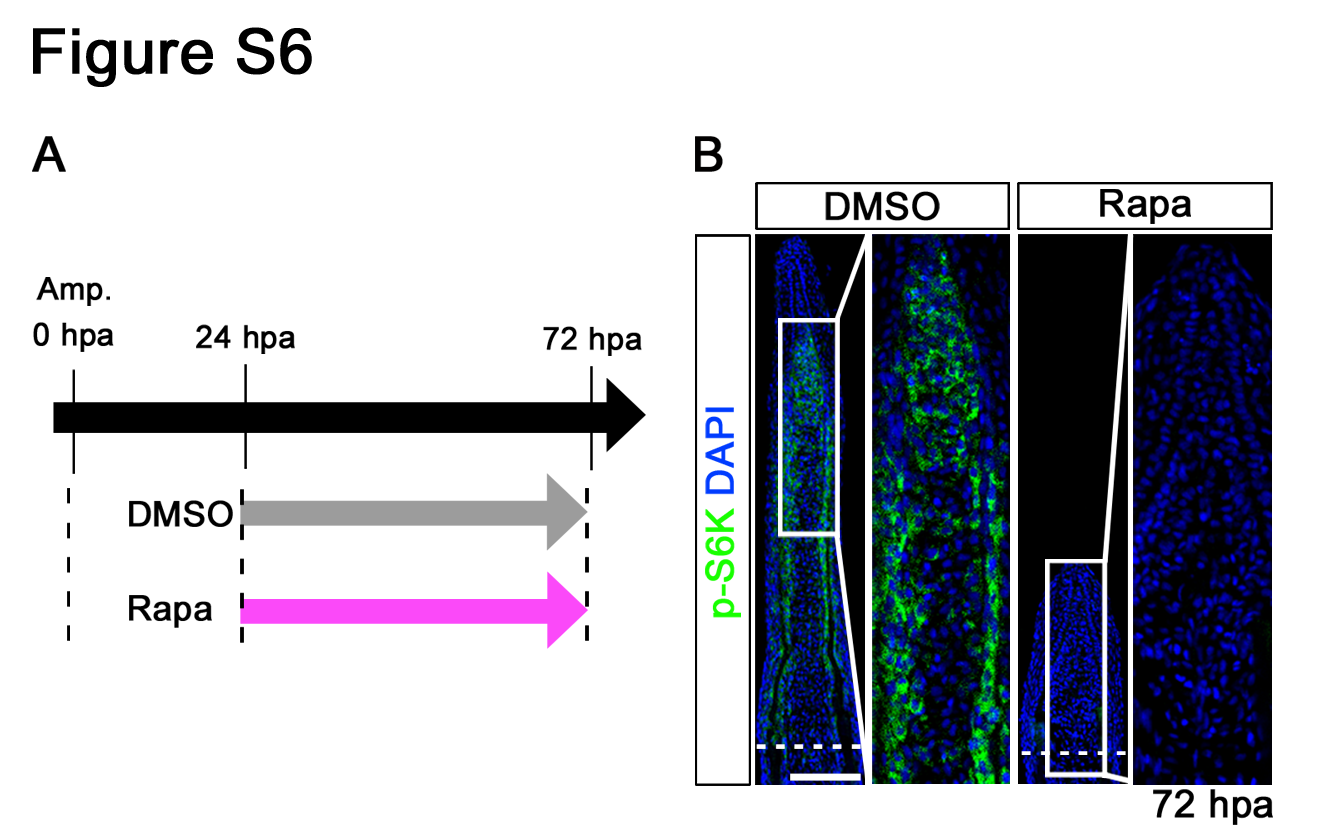

Supplement: Additional file 6: Figure S6. — Distributions of p-S6K in fin regenerates treated with rapamycin from 24 to 72 hpa. (A) Scheme of rapamycin treatment from 24 to 72 hpa. (B) Longitudinal sections of wild-type fin regenerates that were immunohistochemically stained with an antibody against p-S6K (green) at 72 hpa (DMSO, n = 5; Rapa, n = 5). DAPI fluorescent signal (blue) indicates the presence of nuclei. At 72 hpa, the p-S6K fluorescent signal was lost in rapamycin-treated fin regenerates. Dashed white lines indicate the amputation plane. Scale bars: 100 μm. [file 12861_2014_42_MOESM6_ESM.tiff]

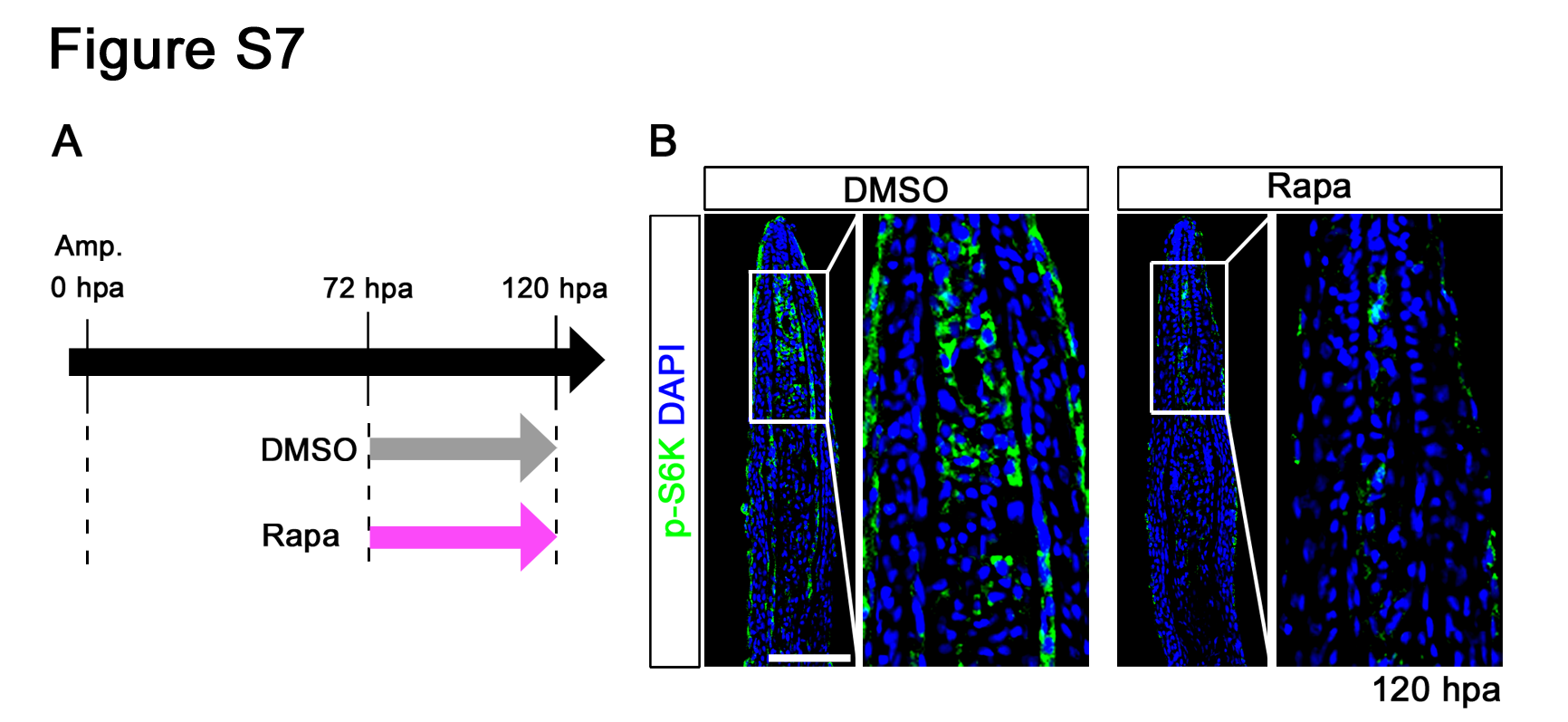

Supplement: Additional file 7: Figure S7. — Distributions of p-S6K in fin regenerates treated with rapamycin from 72 to 120 hpa. (A) Scheme of rapamycin treatment from 72 to 120 hpa. (B) Longitudinal sections of wild-type fin regenerates that were immunohistochemically stained with an antibody against p-S6K (green) at 120 hpa (DMSO, n = 3; Rapa, n = 3). DAPI fluorescent signal (blue) indicates the presence of nuclei. At 120 hpa, the fluorescent signal of p-S6K was markedly reduced by rapamycin treatment. Scale bars: 100 μm. [file 12861_2014_42_MOESM7_ESM.tiff]

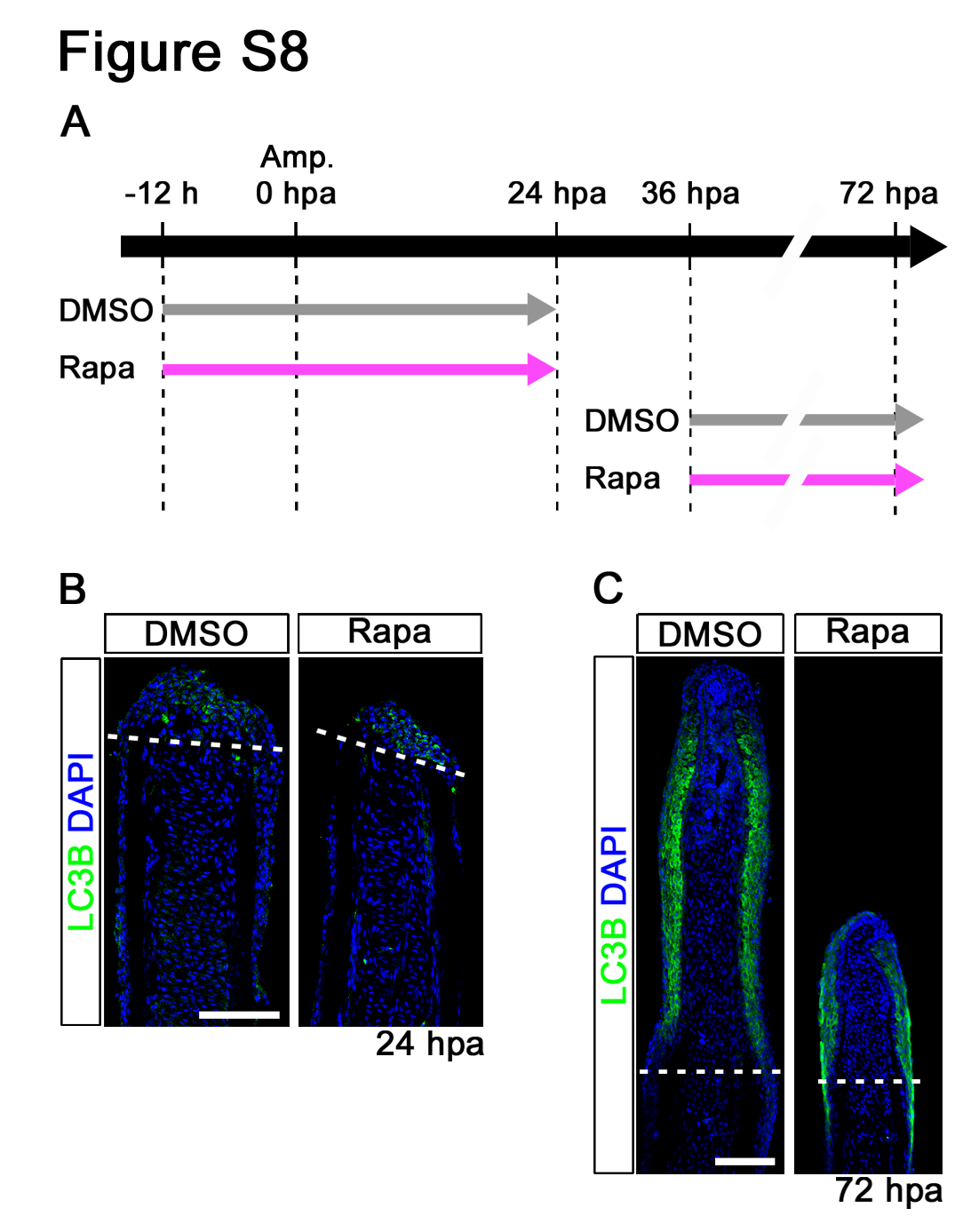

Supplement: Additional file 8: Figure S8. — Distributions of LC3B in rapamycin treated fin regenerates. (A) Scheme of rapamycin treatment from -12 to 24 hpa or from 36 to 72 hpa. (B,C) Longitudinal sections of DMSO or rapamycin treated wild-type fin regenerates that were immunohistochemically stained with an antibody against LC3B (green) at 24 hpa (DMSO, n = 4; Rapa, n = 4) and 72 hpa (DMSO, n = 3; Rapa, n = 3). DAPI fluorescent signal (blue) indicates the presence of nuclei. LC3B is specifically localized in the wound epidermis at 24 and 72 hpa. Dashed white lines indicate the amputation planes. Scale bars: 100 μm. [file 12861_2014_42_MOESM8_ESM.tiff]

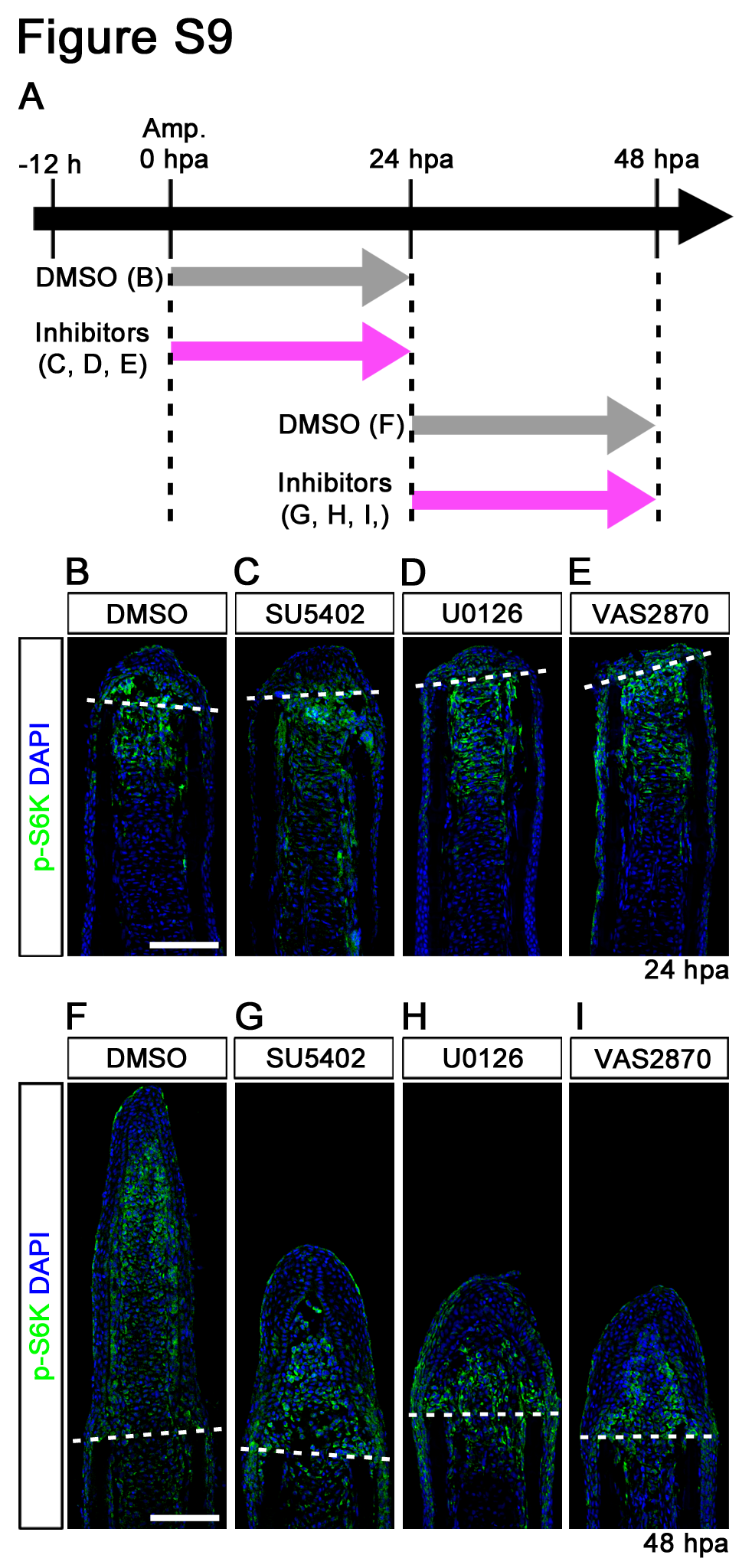

Supplement: Additional file 9: Figure S9. — Fgf, MAPK/Erk, and ROS signaling pathways do not regulate the activation of mTORC1 during fin regeneration. (A) Scheme of inhibitor treatments of SU5402 (a Fgf receptor1 inhibitor), U0126 (a MAPK/Erk inhibitor), and VAS2870 (an NADPH oxidase inhibitor: ROS signaling) during fin regeneration. (B-I) Longitudinal sections of DMSO or inhibitors treated wild-type fin regenerates that were immunohistochemically stained with an antibody against p-S6K (green) at 24 or 48 hpa. DAPI fluorescent signal (blue) indicates the presence of nuclei. The p-S6K signals in SU5402-, U0126-, and VAS2870-treated fins were approximately comparable to those in DMSO-treated fins at both 24 hpa (DMSO, n = 6; SU5402, n = 5; U0126, n = 3; VAS2870, n = 3) and 48 hpa (DMSO, n = 6; SU5402, n = 4; U0126, n = 3; VAS2870, n = 3), even though these inhibitors, at 48 hpa, prevented fin regeneration. Dashed white lines indicate the amputation planes. Scale bars: 100 μm. [file 12861_2014_42_MOESM9_ESM.tiff]
